# Design, Syntheses, and Bioevaluations of Some Novel *N*^2^-Acryloylbenzohydrazides as Chemostimulants and Cytotoxic Agents

**DOI:** 10.3390/medicines8060027

**Published:** 2021-06-03

**Authors:** Kinjal Lakhani, Edgar A. Borrego, Karla G. Cano, Jonathan R. Dimmock, Renato J. Aguilera, Swagatika Das, Praveen K. Roayapalley, Rajendra K. Sharma, Umashankar Das

**Affiliations:** 1 Drug Discovery and Development Research Cluster, College of Pharmacy and Nutrition, University of Saskatchewan, 107 Wiggins Road, Saskatoon, SK S7N 5E5, Canada; kinjal.lakhani@usask.ca (K.L.); dasswagatika@yahoo.co.in (S.D.); rpraveen.sp@usask.ca (P.K.R.); umashankar.usask@gmail.com (U.D.); 2 Department of Biological Sciences, Border Biomedical Research Center, The University of Texas at El Paso, 500 West University Avenue, El Paso, TX 79968-0519, USA; eaborregopu@miners.utep.edu (E.A.B.); Karla.CanoHernandez@UTSouthwestern.edu (K.G.C.); 3 Department of Pathology and Laboratory Medicine, University of Saskatchewan, 107 Wiggins Road, Saskatoon, SK S7N 5E5, Canada; rajendra.sharma@usask.ca

**Keywords:** acryloylhydrazides, cytotoxins, chemosensitization, antineoplastic agents, mitochondrial membrane potential, reactive oxygen species

## Abstract

A series of novel *N*^2^-acryloylhydrazides **1a–m** and a related series of compounds **6a–c** were prepared as potential chemostimulants. In general, these compounds are cytotoxic to human HCT 116 colon cancer cells, as well as human MCF-7 and MDA-MB-231 breast cancer cell lines. A representative compound *N*^1^-(3,4-dimethoxyphenylcarbonyl)-*N*^2^-acryloylhydrazine **1m** sensitized HCT 116 cells to the potent antineoplastic agent 3,5-bis(benzylidene)-4-piperidone **2a**, and also to 5-fluorouracil. A series of compounds was prepared that incorporated some of the molecular features of **2a** and related compounds with various *N*^2^-acryloylhydrazides in series 1. These compounds are potent cytotoxins. Two modes of action of representative compounds are the lowering of mitochondrial membrane potential and increasing the concentration of reactive oxygen species.

## 1. Introduction

There are a number of disadvantages of contemporary anticancer drugs, including their toxicity. Thus, approaches aimed at reducing the amounts of drugs administered, while retaining antineoplastic potency, are important strategies. The approach taken in this study is to examine whether the prior administration of a candidate chemostimulant evokes a synergistic biological response. In the current investigation, the decision was made to design chemostimulants ab initio that have the potential of being cytotoxic. These compounds are structurally divergent from current anticancer drugs and, hence, may be toxic to neoplasms that are resistant to established anticancer drugs.

**Figure 1:**
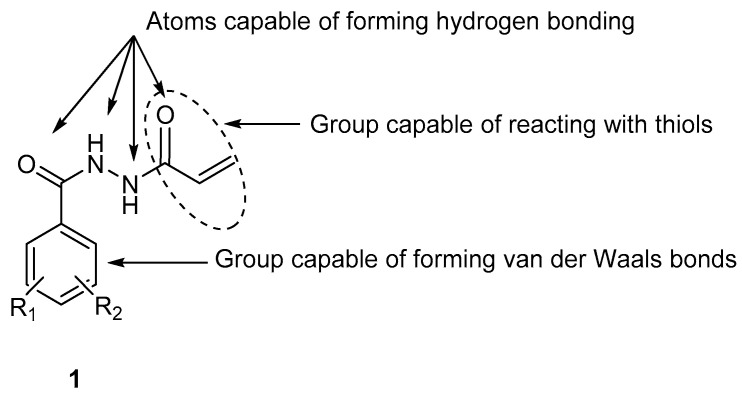
Atoms and groups in series **1** which are capable of bonding with cellular constituents.

The compounds in series 1 were designed as chemostimulants, and the reasons for the presence of different atoms and groups are indicated in Figure 1. Thus an aryl ring will enable van der Waals bonding to take place with cellular constituents. The secondary amino groups (NH) can act as hydrogen donor atoms, while the oxygen atoms are hydrogen bond acceptors. The terminal α,β-unsaturated keto group was incorporated into series **1**, since it has a marked affinity for thiol groups [1]. The chosen cytotoxin is a member of series **2**, as these compounds have previously demonstrated potent antineoplastic properties [2].

**Figure 2:**
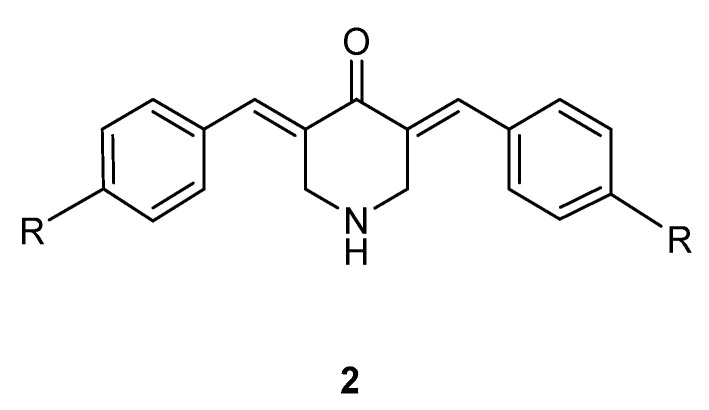
Cytotoxic 3,5-bis(benzylidene)-4-piperidones **2**.

In summary, the two principal objectives of this study are as follows: First, to synthesize and evaluate the potential of series **1** as novel cytotoxic agents, and to determine whether a representative compound would chemosensitize a tumor cell line to a 4-piperidone in series **2** (Figure 2). Second, if the bioactivity of one or more compounds in series **1** displayed promising antineoplastic properties, then evaluations using additional neoplastic cell lines would be undertaken. Furthermore, a combination of the structural features of some of the compounds in series **1** and **2** may lead to a novel series of candidate antineoplastic agents. The determination of some of the modes of action of representative compounds that display cytotoxicity was also planned.

## 2. Materials and Methods

### 2.1. Synthesis of Compounds

#### 2.1.1. Synthesis of *N*^1^-aroyl-*N*^2^-acryloylhydrazides **1a**–**m**

The ^1^H and ^13^C nuclear magnetic resonance (NMR) spectra were determined using a Bruker Avance AMX 500 FT spectrometer equipped with a BBO probe. The chemical shifts were recorded in ppm. Mass spectra were obtained using a QSTAR XL hybrid LC/MS/MS instrument. Melting points were obtained with a Gallenkamp instrument, recorded in degrees Celsius, and are uncorrected. Thin-layer chromatography (TLC) was undertaken with silica-gel-F_254_-precoated plates when monitoring the chemical reactions.

The synthesis of the intermediate ethyl esters 4a–m and the hydrazides **5a**–**m** are presented in the Supplementary Schemes S1 and S2.

The general method for the preparation of **1a**–**m** is as follows: A benzohydrazide (1 g, 7.34 mmol) was added to a solution of sodium bicarbonate (0.678 g, 8.07 mmol) in water (7.5 mL), and the reaction vessel was placed in an ice bath. After 0.25 h, a solution of acryloyl chloride (0.70 g, 7.70 mmol) in chloroform (7.5 mL) was added dropwise with constant stirring to the suspension of the acid hydrazide, and the mixture was stirred at ice bath temperatures for 1 h. The reaction was monitored via TLC using a solvent system of chloroform and methanol (9.5:0.5). After the solvent was removed in vacuo, the residue was collected, washed with ice cold water, and dried [3]. The product was dissolved in ethyl acetate (20 mL) and washed twice with hydrochloric acid (2N, 2 × 10 mL). The organic phase was separated, washed with brine solution (10 mL), and dried, and after the evaporation of the solvent, the product obtained was recrystallized from ethanol (95%).

##### *N*^2^-Acryloylbenzohydrazide **1a**

Yield = 86%; m.p. 177–179 °C (lit. m.p. 175–178 °C) [4]; ^1^H NMR (500 MHz, DMSO-*d*_6_) δ ppm 10.34 (d, J = 109.3 Hz, 2H, CONH), 7.88 (m, 2H, Ar-H),7.59 (m, 1H, Ar-H), 7.50 (m, 2H, Ar-H), 6.35 (q, J = 17.17 Hz, 10.2 Hz, 1H, =CH), 6.23 (dd, J = 17.17 Hz, 2.05 Hz, 1H, =CH), 5.76 (dd, J = 10.2 Hz, 2.06 Hz, 1H, =CH). ^13^C NMR (DMSO-d_6_): 165.40, 163.87, 132.42, 131.84, 129.36, 128.47, 127.43, 126.93. MS (ESI): *m*/*z* 191.08 [M+H] ^+^.

##### *N*^2^-Acryloyl-4-fluorobenzohydrazide **1b**

Yield = 79%; m.p.176–179 °C; ^1^H NMR (500 MHz, DMSO-*d*_6_) δ ppm 10.51 (d, J = 1.185 Hz, 1H, CONH), 10.22 (d, J = 1.206 Hz, 1H, CONH), 7.96 (m, 2H, Ar-H), 7.35 (m, 2H, Ar-H), 6.34 (q, J = 17.11 Hz,10.2 Hz, 1H, =CH),6.23 (dd, J = 17.17 Hz, 2.04 Hz, 1H, =CH), 5.76 (dd, J = 10.2 Hz, 2.07 Hz, 1H, =CH). The principal peak [M+H] ^+^ was not found in the mass spectra however the fragment peak was found. MS (ESI): *m*/*z* 105.95 [M+H] ^+^.

##### *N*^2^-Acryloyl-4-bromobenzohydrazide **1c**

Yield = 70%; m.p. 256–259 °C; ^1^H NMR (500 MHz, DMSO-*d*_6_) δ ppm 10.58 (d, J = 1.46 Hz, 1H, CONH), 10.25 (d, J = 1.45 Hz, 1H, CONH), 7.81 (m, 2H, Ar-H), 7.74 (m, 2H, Ar-H), 6.34 (q, J = 17.3 Hz, 10.16 Hz, 1H, =CH), 6.23 (dd, J = 17.07 Hz, 2.10 Hz, 1H, =CH), 5.76 (dd, J = 10.21 Hz, 2.06 Hz, 1H, =CH). MS (ESI): *m*/*z* 290.97 [M+Na] ^+^, 268.99 [M+H] ^+^.

##### *N*^2^-Acryloyl-4-chlorobenzohydrazide **1d**

Yield = 73%; m.p. 233–235 °C; ^1^H NMR (500 MHz, DMSO-*d*_6_) δ ppm 10.58 (d, J = 1.45 Hz, 1H, CONH), 10.25 (d, J = 1.49 Hz, 1H, CONH), 7.90 (m, 2H, Ar-H), 7.6 (m, 2H, Ar-H), 6.34 (q, J = 17.15 Hz, 10.22 Hz, 1H, =CH), 6.23 (dd, J = 17.15 Hz, 2.09 Hz, 1H, =CH), 5.76 (dd, J = 10.18 Hz, 2.06 Hz, 1H, =CH). MS (ESI): *m*/*z* 247.02 [M+Na] ^+^, 225.04 [M+H] ^+^.

##### *N*^2^-Acryloyl-2-chlorobenzohydrazide **1e**

Yield = 67%; m.p. 165–167 °C; ^1^H NMR (500 MHz, DMSO-*d*_6_) δ ppm 10.41 (brs, 2H, CONH), 7.52 (m, 3H, Ar-H), 7.44 (m, 1H, Ar-H), 6.33 (q, J = 17.15 Hz, 10.07 Hz, 1H, =CH), 6.23 (dd, J = 17.13 Hz, 2.13 Hz, 1H, =CH), 5.76 (dd, J =10.09 Hz, 2.13 Hz, 1H, =CH). MS (ESI): *m*/*z* 262.85 [M+K] ^+^, 247.02 [M+Na] ^+^, 225.04 [M+H] ^+^.

##### *N*^2^-Acryloyl-3-chlorobenzohydrazide **1f**

Yield = 86%; m.p. 155–158 °C; ^1^H NMR (500 MHz, DMSO-*d*_6_) δ ppm 10.62 (brs, 1H, CONH), 10.28 (s, 1H, CONH),7.91 (t, 1H, Ar-H), 7.84 (m, 1H, Ar-H), 7.67 (m, 1H, Ar-H), 7.56 (t, 1H, Ar-H), 6.34 (q, J = 17.15 Hz, 10.20 Hz, 1H, =CH), 6.23 (dd, J = 17.16 Hz, 2.06 Hz, 1H, =CH), 5.76 (dd, J = 10.21 Hz, 2.06 Hz, 1H, =CH). MS (ESI): *m*/*z* 247.02 [M+Na] ^+^, 225.04 [M+H] ^+^.

##### *N*^2^-Acryloyl-3,4-dichlorobenzohydrazide **1g**

Yield = 78%; m.p. 172–175 °C; ^1^H NMR (500 MHz, DMSO-*d*_6_) δ ppm 10.71 (s, 1H, CONH), 10.32 (s, 1H, CONH), 8.11 (d, J = 1.91 Hz, 1H, Ar-H), 7.87 (dd, J = 8.39 Hz, 2.00 Hz, 1H, Ar-H), 7.81 (d, J = 8.37 Hz, 1H, Ar-H), 6.35 (q, J = 17.18 Hz, 10.24 Hz, 1H, =CH), 6.23 (dd, J = 17.16 Hz, 2.06 Hz, 1H, =CH), 5.76 (dd, J = 10.16 Hz, 2.04 Hz, 1H, =CH). MS (ESI): *m*/*z* 280.98 [M+Na] ^+^, 259.00 [M+H] ^+^.

##### *N*^2^-Acryloyl-2,5-dichlorobenzohydrazide **1h**

Yield = 69%; m.p. 207–210 °C; ^1^H NMR (500 MHz, DMSO-*d*_6_) δ ppm 10.60 (d, J = 1.93 Hz, 1H, CONH), 10.45 (d, J = 1.93 Hz, 1H, CONH), 7.60 (m, 2H, Ar-H), 7.53 (m, 1H, Ar-H), 6.33 (q, J = 17.11 Hz, 10.09 Hz, 1H, =CH), 6.24 (dd, J = 17.14 Hz, 2.14 Hz, 1H, =CH), 5.76 (dd, J = 10.06 Hz, 2.16 Hz, 1H, =CH). MS (ESI): *m*/*z* 259.00 [M+H] ^+^.

##### *N*^2^-Acryloyl-3-methylbenzohydrazide **1i**

Yield = 87%; m.p. 138–140 °C; ^1^H NMR (500 MHz, DMSO-*d*_6_) δ ppm 10.41 (d, J = 1.41 Hz, 1H, CONH), 10.20 (d, J = 1.77 Hz, 1H, CONH), 7.67 (m, 2H, Ar-H), 7.39 (m, 1H, Ar-H), 6.34 (q, J=17.13 Hz, 10.19 Hz, 1H, =CH), 6.22 (dd, J=17.12 Hz, 2.09 Hz, 1H, =CH), 5.75 (dd, J = 10.21 Hz, 2.09 Hz, 1H, =CH), 2.37 (s, 3H, CH_3_). MS (ESI): *m*/*z* 227.08 [M+Na] ^+^, 205.09 [M+H] ^+^.

##### *N*^2^-Acryloyl-2-methylbenzohydrazide **1j**

Yield = 82%; m.p. 151–153 °C; ^1^H NMR (500 MHz, DMSO-*d*_6_) δ ppm 10.18 (s, 2H, CONH), 7.38 (m, 2H, Ar-H), 7.27 (m, 2H, Ar-H), 6.33 (q, J = 17.14 Hz, 10.08 Hz, 1H, =CH), 6.23 (dd, J = 17.15 Hz, 2.16 Hz, 1H, =CH), 5.75 (dd, J = 10.09 Hz, 2.16 Hz, 1H, =CH), 2.39 (s, 3H, CH_3_). MS (ESI): *m*/*z* 227.08 [M+Na] ^+^, 205.09 [M+H] ^+^.

##### *N*^2^-Acryloyl-4-methylbenzohydrazide **1k**

Yield = 79%; m.p. 157–160 °C; ^1^H NMR (500 MHz, DMSO-*d*_6_) δ ppm 10.29 (d, J = 85.67, 2H, CONH), 7.79 (d, J = 8.17 Hz, 2H, Ar-H), 7.30 (d, J = 8.00 Hz, 2H, Ar-H), 6.34 (q, J = 17.16 Hz, 10.20 Hz, 1H, =CH), 6.21 (dd, J = 17.13 Hz, 2.07 Hz, 1H, =CH), 5.75 (dd, J = 10.16 Hz, 2.10 Hz, 1H, =CH), 2.37 (s, 3H, CH_3_). MS (ESI): *m*/*z* 205.09 [M+H] ^+^.

##### *N*^2^-Acryloyl-4-methoxylbenzohydrazide **1l**

Yield = 85%; m.p. 165–166 °C; ^1^H NMR (500 MHz, DMSO-*d*_6_) δ ppm 10.24 (d, J = 83.79 Hz, 2H, CONH), 7.87 (dd, J = 8.85 Hz, J = 4.95, 2H, Ar-H), 7.03 (dd, J = 8.85, J = 5.04 Hz, 2H, Ar-H), 6.34 (q, J = 17.16 Hz, 10.22 Hz, 1H, =CH), 6.22 (dd, J = 17.13 Hz, 2.10 Hz, 1H, =CH), 5.74 (dd, J = 10.22 Hz, 2.05 Hz, 1H, =CH), 3.82 (s, 3H, OCH_3_). MS (ESI): *m*/*z* 243.07 [M+Na] ^+^, 221.09 [M+H] ^+^.

##### *N*^2^-Acryloyl-3,4-dimethoxylbenzohydrazide **1m**

Yield = 75%; m.p. 163–165 °C; ^1^H NMR (500 MHz, DMSO-*d*_6_) δ ppm 10.25 (brs, 2H, CONH), 7.53 (dd, J = 8.38 Hz, 1H, Ar-H), 7.47 (d, J = 2.00 Hz, 1H, Ar-H), 7.06 (d, J = 8.53 Hz, 1H, Ar-H), 6.35 (q, J = 17.13 Hz, 10.24 Hz, 1H, =CH), 6.22 (dd, J = 17.12 Hz, 2.08 Hz, 1H, =CH), 5.75 (dd, J = 10.22 Hz, 2.07 Hz, 1H, =CH), 3.81 (d, J = 5.26 Hz, 6H, 2xOCH_3_). MS (ESI): *m*/*z* 273.08 [M+Na] ^+^, 251.10 [M+H] ^+^.

#### 2.1.2. Synthesis of the *N*^2^-Acylbenzohydrazides **6a**–**c**

Propionic anhydride (0.95 mL, 7.34 mmol) was added dropwise to a solution of benzohydrazide (1 g, 7.34 mmol) in ethanol (5 mL), and the mixture was stirred at room temperature for 0.25 h. The product was triturated several times with diethyl ether, filtered, and recrystallized from ethanol (95%) to produce **6a**.

##### *N*^2^-Propionylbenzohydrazide **6a**

Yield = 87%; m.p.106–107 °C (lit. m.p.110 °C) [5]; ^1^H NMR (500 MHz, DMSO-*d*_6_) δ ppm 10.29 (s, 1H, CONH), 9.84 (s, 1H, CONH), 7.86 (m, 2H, Ar-H), 7.58 (m, 1H, Ar-H), 7.50 (t, 2H, Ar-H), 2.19 (q, 2H, CH_2_), 1.06 (t, 3H, CH_3_). ^13^C NMR (DMSO-d_6_): 172.68, 165.77, 132.67, 131.92, 128.46, 127.54, 26.70, 9.80. MS (ESI): *m*/*z* 215.07 [M+Na] ^+^, 193.09 [M+H] ^+^.

The analogue **6b** was prepared in an identical manner, using acetic anhydride in place of propionic anhydride.

##### *N*^2^-Acetylbenzohydrazide **6b**

Yield = 83%; m.p.171–172 °C (lit. m.p. 174 °C) [5]; ^1^H NMR (500 MHz, DMSO-*d*_6_) δ ppm 10.28 (s, 1H, CONH), 9.88 (s, 1H, CONH), 7.86 (m, 2H, Ar-H), 7.58 (m, 1H, Ar-H), 7.50 (m, 2H, Ar-H), 1.91 (s, 3H, CH_3_). ^13^C NMR (DMSO-d_6_): 168.84, 165.74, 132.60, 131.96, 128.60, 127.54, 20.72. MS (ESI): *m*/*z* 217.04 [M+K] ^+^, 179.07 [M+H] ^+^.

A solution of benzohydrazide (0.72 g, 0.005 mol) and *N*^1^-phenyl-*N*^2^-acryloylhydrazine **1a** (1 g, 0.005 mol) dissolved in ethanol (10 mL) was heated under reflux for 24 h. The reaction was monitored via TLC using a solvent system of chloroform and methanol (9.5:0.5). The solvent was removed in vacuo and the residue was recrystallized from ethanol (95%) to produce **6c**.

##### *N*^2^-[3-(Benzoylaminoamino)-1-oxopropyl]-*N*^1^-benzoylhydrazine **6c**

Yield = 69%; m.p. 193–195 °C; ^1^H NMR (500 MHz, DMSO-*d*_6_) δ ppm 10.36 (s, 1H, CONH), 10.05 (d, J = 6.23 Hz, 1H, CONH), 9.99 (s, 1H, CONH), 7.85 (q, J = 17.95 Hz, 7.45 Hz, 4H-Ar), 7.50 (m, 6H, Ar-H), 5.39 (q, 1H, NH), 3.06 (q, 2H, CH_2_), 2.42 (t, 2H, CH_2_). MS (ESI): *m*/*z* 327.14 [M+H] ^+^.

#### 2.1.3. Synthesis of the 3,5-bis(benzylidene)-4-piperidones **2a**–**f**

The compounds in series **2** have been prepared previously, and were synthesized by the method of [6]. The 4-piperidone **2a**, which was used in the biological experiments and in the synthesis of **7a**, was purified via crystallization from ethanol (95%). The analogs **2b**–**f** were isolated and used without purification in the synthesis of **7b**–**f**. The ^1^HNMR spectra of **2a**–**f** were consistent with the structures proposed, and these data are presented in the Supplemental Section S3.

#### 2.1.4. Synthesis of the *N*-alkyl-3,5-bis(benzylidene)-4-piperidones **7a**–**g**

A mixture of 3,5-bis(benzylidene)-4-piperidone (0.0036 mol), *N*^1^-aroyl-*N*^2^-acryloylhydrazide (0.011 mol), triethylamine (0.18 mol), dichloromethane (10 mL), and methanol (5 mL) was heated at reflux temperature for 24 h. The reaction was monitored via TLC using a solvent system of chloroform and methanol (9.5:0.5). On completion of the reaction, the solvents were removed in vacuo and the products were crystallized from ethanol (95%).

##### 3,5-Bis(benzylidene)-1-[3-(phenylcarbonylaminoamino)-3-oxo-1-propyl]-4-piperidone **7a**

Yield = 81%; m.p. 154–156 °C; ^1^H NMR (500 MHz, DMSO-*d*_6_) δ ppm 10.34 (s, 1H, CONH), 9.92 (s, 1H, CONH), 7.83 (d, J = 7.82, 2H, Ar-H), 7.62 (s, 2H, =CH), 7.55 (d, J = 8.45 Hz, 5H, Ar-H), 7.48 (dd, J = 7.79, 5H, Ar-H), 7.44 (m, 3H, Ar-H), 3.88 (s, 4H, piperidyl H), 2.87 (t, J = 7.02 Hz, J = 14.05 Hz, 2H, CH_2_), 2.39 (t, J = 7.03 Hz, J = 14.07 Hz, 2H, CH_2_). ^13^C NMR (DMSO-d_6_): 170.22, 166.21, 165.34, 134.85, 134.61, 133.74, 132.41, 131.06, 130.62,129.33, 128.83, 128.45, 127.43, 54.06, 52.75, 32.54, 31.17. MS (ESI): *m*/*z* 466.21 [M+H] ^+^.

##### 3,5-Bis(benzylidene)-1-[3-(3,4-dimethoxylphenylcarbonylaminoamino)-3-oxo-1-propyl]-4-piperidone **7b**

Yield = 86%; m.p. 151–154 °C; ^1^H NMR (500 MHz, DMSO-*d*_6_) δ ppm 10.20 (s, 1H, CONH), 9.86 (s, 1H, CONH), 7.62 (s, 2H, =CH), 7.55 (d, J = 7.31 Hz, 4H, Ar-H), 7.5 (m, 6H, Ar-H), 7.43 (m, 2H, Ar-H), 7.02 (d, J = 8.51 Hz, 1H, Ar-H), 3.88 (s, 4H, piperidyl H), 3.79 (d, J = 13.42 Hz, 6H, 2xOCH_3_), 2.87 (t, J = 6.85 Hz, J = 13.70 Hz, 2H, CH_2_), 2.39 (t, J = 6.94 Hz, J = 13.91 Hz, 2H, CH_2_). MS (ESI): *m*/*z* 526.23 [M+H] ^+^.

##### 3,5-Bis(4-chlorobenzylidene)-1-[3-(3,4-dimethoxyphenylcarbonylaminoamino)-3-oxo-1-propyl]-4-piperidone **7c**

Yield = 76%; m.p. 200–202 °C; ^1^H NMR (500 MHz, DMSO-*d*_6_) δ ppm 10.21 (s, 1H, CONH), 9.86 (s, 1H, CONH), 7.59 (s, 2H, =CH), 7.55 (q, J = 21.20 Hz, 8H, Ar-H), 7.48 (dd, J = 8.47, 1H, Ar-H), 7.43 (d, J = 1.86 Hz, 1H, Ar-H), 7.03 (d, J = 8.52 Hz, 1H, Ar-H), 3.85 (s, 4H, piperidyl H), 3.79 (d, J = 12.07 Hz, 6H, 2xOCH_3_), 2.87 (t, J = 6.88 Hz, 2H, CH_2_), 2.34 (t, J = 6.83 Hz, 2H, CH_2_). MS (ESI): *m*/*z* 594.15, 595.15 [M+H] ^+^, 596.15 [M+2H] ^+^.

##### 3,5-Bis(4-fluorobenzylidene)-1-[3-(3,4-dimethoxyphenylcarbonylaminoamino)-3-oxo-1-propyl]-4-piperidone **7d**

Yield = 80%; m.p.198–200 °C; ^1^H NMR (500 MHz, DMSO-*d*_6_) δ ppm 10.21 (s, 1H, CONH), 9.86 (s, 1H, CONH), {m, 6H (4H, Ar-H; 2H, =CH)}, 7.5 (dd, J = 8.42 Hz, 1H, Ar-H), 7.43 (d, J = 1.85 Hz, 1H, Ar-H), 7.31 (t, J = 8.81 Hz, 4H, Ar-H), 7.03 (d, J = 8.56 Hz, 1H, Ar-H), 3.85 (s, 4H, piperidyl H), 3.79 (d, J = 12.47 Hz, 6H, 2xOCH_3_), 2.87 (t, J = 6.94 Hz, 2H, CH_2_), 2.4 (t, J = 6.91 Hz, 2H, CH_2_). MS (ESI): *m*/*z* 562.21 [M+H] ^+^.

##### 3,5-Bis(4-nitrobenzylidene)-1-[3-(3,4-dimethoxyphenylcarbonylaminoamino)-3-oxo-1-propyl]-4-piperidone **7e**

Yield = 71%; m.p. 153–158 °C; ^1^H NMR (500 MHz, DMSO-*d*_6_) δ ppm 10.22 (s, 1H, CONH), 9.87 (s, 1H, CONH), 8.29 (d, J = 8.76 Hz, 4H, Ar-H), 7.82 (d, J = 8.78 Hz, 2H, Ar-H), 7.77 (d, J = 8.78 Hz, 2H, Ar-H), 7.69 (d, J = 20.10 Hz, 2H, =CH), 7.48 (dd, J = 8.45 Hz, 10.38 Hz, 1H, Ar-H), 7.43 (d, J = 1.84 Hz, 1H, Ar-H), 7.02 (d, J = 8.55 Hz, 1H, Ar-H), 4.02 (s, 2H, piperidyl H), 3.92 (s, 2H, piperidyl H), 3.79 (d, J = 13.91 Hz, 6H, 2xOCH_3_), 2.88 (t, J = 6.78 Hz, 13.56 Hz, 2H, CH_2_), 2.39 (t, J = 6.78 Hz, 13.58 Hz, 2H, CH_2_). MS (ESI): *m*/*z* 616.20 [M+H] ^+^.

##### 3,5-Bis(4-methoxybenzylidene)-1-[3-(3,4-dimethoxyphenylcarbonylaminoamino)-3-oxo-1-propyl]-4-piperidone **7f**

Yield = 75%; m.p. 175–178 °C; ^1^H NMR (500 MHz, DMSO-*d*_6_) δ ppm 10.21 (s, 1H, CONH), 9.88 (s, 1H, CONH), 7.56 (s, 2H, =CH), 7.50 (d, J = 8.88 Hz, 4H, Ar-H), 7.45 (m, 2H, Ar-H), 7.036 (m, 4H, Ar-H), 7.02 (s, 1H, Ar-H), 3.84 (s, 3H, OCH_3_), 3.81 (s, 6H, 2xOCH_3_), 3.80 (s, 4H, piperidyl H), 3.77 (s, 3H, OCH_3_), 2.88 (t, J = 6.9 Hz, 13.80 Hz, 2H, CH_2_), 2.41 (t, J = 6.88 Hz, 13.76 Hz, 2H, CH_2_). MS (ESI): *m*/*z* 586.25 [M+H] ^+^.

##### 3,5-Bis(4-methylbenzylidene)-1-[3-(3,4-dimethoxyphenylcarbonylaminoamino)-3-oxo-1-propyl]-4-piperidone **7g**

Yield = 82%; m.p. 190–192°C; ^1^H NMR (500 MHz, DMSO-*d*_6_) δ ppm 10.20 (s, 1H, CONH), 9.86 (s, 1H, CONH), 7.58 (s, 2H, =CH), 7.48 (dd, J = 8.37 Hz, 1H, Ar-H), 7.43 (m, 5H, Ar-H), 7.3 (d, J = 8.09 Hz, 4H, Ar-H), 7.02 (d, J = 8.54 Hz, 1H, Ar-H), 3.85 (s, 4H, piperidyl H), 3.79 (d, J = 13.07 Hz, 6H, 2xOCH_3_), 2.86 (t, J = 7.04 Hz, 2H, CH_2_), 2.38 (t, J = 7.14 Hz, 2H, CH_2_), 2.35 (s, 6H, CH_3_). MS (ESI): *m*/*z* 554.26 [M+H] ^+^.

### 2.2. Bioevaluations

#### 2.2.1. Cytotoxicity Assays

Evaluation of the cytotoxic properties of the compounds presented in Table 1, Table 2, Table 3 and Table 4 was undertaken using the procedure outlined in [7]. In brief, the HCT 116 cells were obtained from the ATCC and cultured in McCoy’s 5A medium. The CRL 1790 cells were obtained from the ATCC and cultured in Minimum Essential Medium. The MCF-7 and MDA-MBA-231 cells were obtained from the Saskatchewan Cancer Agency and cultured in DMEM. In each assay the culture medium was supplemented with 10% fetal bovine serum and 1% penicillin–streptomycin antibiotics. The time of the bioassays was 48 h, unless indicated otherwise. All determinations were carried out in triplicate on three different occasions.

The procedure of Santiago-Vázquez et al. was followed in the case of the bioevaluations presented in Table 5 [8]. In brief, the compounds were incubated for 24 h with the neoplasms, using DMEM and RPMI media. Three independent experiments were carried out.

#### 2.2.2. Evaluation of **1l** on the Mitochondrial Membrane Potential of Ramos Cells

The effect of **1l** on the mitochondrial membrane potential of Ramos cells was determined using the procedure laid out in [9]. In brief, the cells were treated with **1l** for 5 h, stained with the JC-1 dye, and then the results were analyzed by flow cytometry.

#### 2.2.3. Effect of Compounds on the Mitochondrial Membrane Potential of HCT 116 cells

The method of Crowley et al. was employed in order to ascertain the effects of **1m**, **7a**, **e**, **g**, and **5-FU** on the mitochondrial membrane potential of HCT 116 cells [10]. In brief, the dye tetramethylrhodamine ethyl ester (TMRE) was used, and the IC50 values of the compounds were added to the HCT 116 cells and incubated for 48 h. The results are presented in Figure 4.

#### 2.2.4. Evaluation of the ROS levels in HCT 116 Cells

In this assay, the dye 2,7-dichlorofluorescein diacetate (DCF-DA) was used, and the effects of **1m**, **7a**, **e**, **g**, and **5-FU** on the ROS levels in HCT 116 cells were determined by the method of [11]. The results are presented in Figure 5.

### 2.3. Quantitative Structure-Activity Relationships

The σ, π and MR constants were obtained from [12]. The correlation analyses were undertaken using a commercial package [13].

## 3. Results

**Scheme 1:**
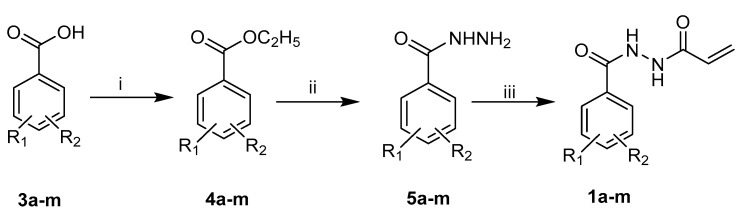
Synthesis of the compounds in series **1**, **3**–**5**. The reagents used are as follows: i = H_2_SO_4_/C_2_H_5_OH; ii = H_2_NNH_2_/C_2_H_5_OH; iii = CH_2_=CHCOCl/CHCl_3_/NaHCO_3_. The aryl substituents are as follows: **a:** R^1^ = R^2^ = H; **b**: R^1^ = 4-F, R^2^ = H; **c**: R^1^ = 4-Br, R^2^ = H; **d**: R^1^ = 4-Cl, R^2^ = H; **e:** R^1^ = 2-Cl, R^2^ = H; **f**: R^1^ = 3-Cl, R^2^ =H; **g**: R^1^ = 3-Cl, R^2^ = 4-Cl; **h**: R^1^ = 2-Cl, R^2^ = 5-Cl; **i:** R^1^ = 3-CH_3_, R^2^ = H; **j:** R^1^ = 2-CH_3_, R^2^ = H; **k:** R^1^ = 4-CH_3_, R^2^ = H; **l:** R^1^ = 4-OCH_3_, R^2^ = H; **m:** R^1^ = 3-OCH_3_, R^2^ = 4-OCH_3_.

**Scheme 2:**
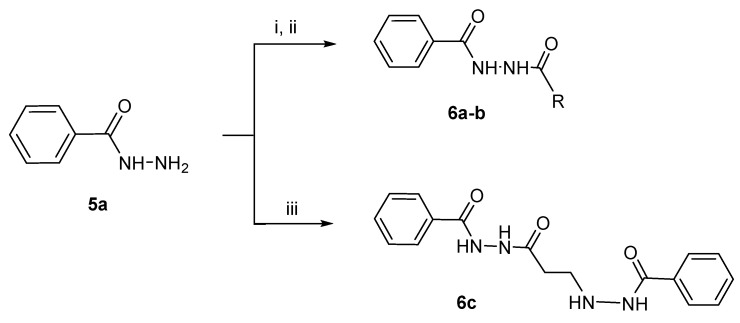
Synthesis of **6a**–**c**. The reagents used are as follows: i = (C_2_H_5_CO)_2_O for **6a**, ii = (CH_3_CO)_2_O for **6b**, and iii = **1a** for **6c**. X: The alkyl groups are as follows: **a** = C_2_H_5_; **b** = CH_3_.

**Scheme 3:**
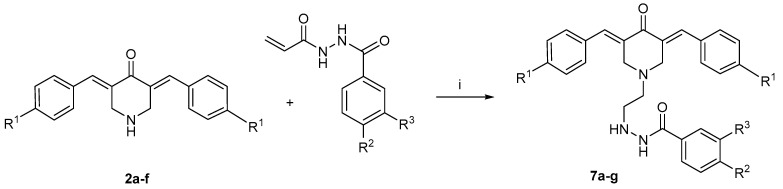
Synthesis of 7a–g. The reagents used are as follows: i = N(C_2_H_5_)_3,_ CH_2_Cl_2,_ CH_3_OH. The aryl substituents in series **2** are as follows: **a**: R^1^ = H; **b**: R^1^ = Cl; **c**: R^1^ = F; **d**: R^1^ = NO_2_; **e**: R^1^ = OCH_3_; **f**: R^1^ = CH_3_. In series **7**, the aryl substituents are **a:** R^1^ = R^2^ = R^3^ = H; **b:** R^1^ = H, R^2^ = R^3^ = OCH_3_; **c:** R^1^ = Cl, R^2^ = R^3^ = OCH_3_; **d:** R^1^ = F, R^2^ = R^3^ = OCH_3_; **e:** R^1^ = NO_2,_ R^2^ = R^3^ = OCH_3_; **f:** R^1^ = R^2^ = R^3^ = OCH_3_; **g:** R^1^ = CH_3_, R^2^ = R^3^ = OCH_3_.

The compounds in series **1** were prepared according to the methodology presented in Scheme 1, whereby a variety of benzoic acids **3** was converted into the corresponding ethyl esters **4**, which gave rise to the related hydrazides **5**. Details of the syntheses of the compounds in series **4** and **5** are presented in the Supplemental section. Reaction of the hydrazides **5** with acryloyl chloride produced the desired compounds **1a**–**m**. The related hydrazides **6a**–**c** were prepared according to the methodology in Scheme 2, while the *N*-alkyl-4-piperidones **7** were synthesized via the procedure presented in Scheme 3 vide infra.

**Figure 3:**
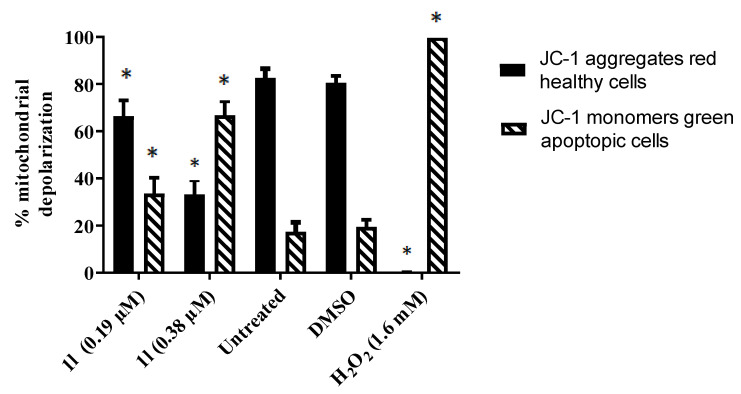
Evaluation of **1l** on the mitochondrial membrane potential of Ramos cells. The asterisks indicate a statistically significant difference (*p* < 0.05) from solvent-treated (DMSO) cells.

**Figure 4:**
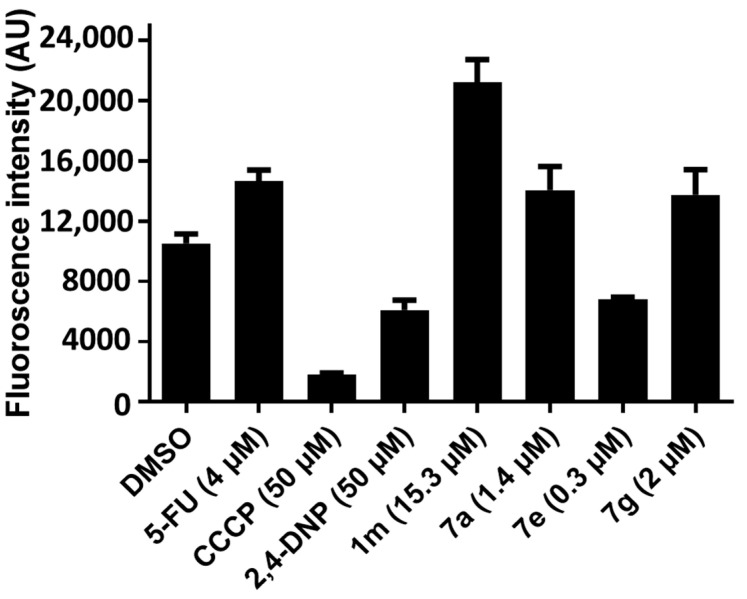
Evaluation of **1m**, **7a**, **e**, and **g** on the mitochondrial membrane potential of HCT 116 cells.

**Figure 5:**
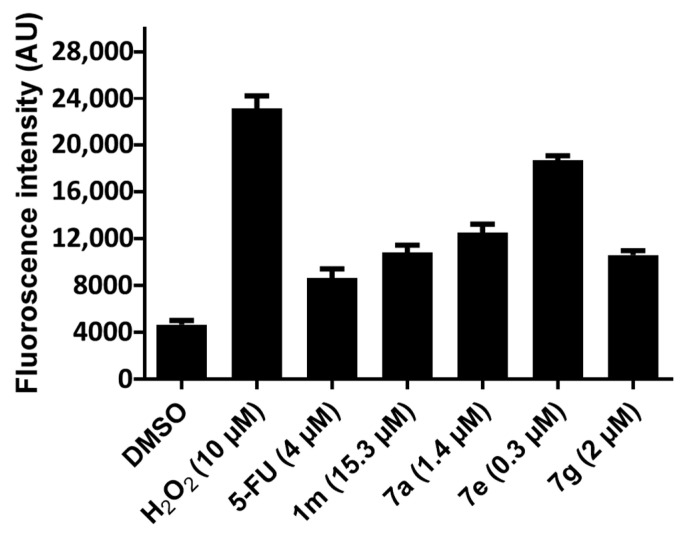
Evaluation of **1m**, **7a**, **e**, and **g** on the production of reactive oxygen species in HCT 116 cells.

The hydrazides in series **1** and **6** were initially screened against human HCT 116 colon cancer cells as well as MCF-7 and MDA-MB-231 breast neoplasms, using concentrations of 10 µM and 100 µM. These data are presented in Table 1. Quantitation of the cytotoxicity of selected compounds towards HCT 116, MCF-7, and human CRL 1790 non-malignant colon cells is given in Table 2. Experimentation designed to evaluate whether **1m** is a chemosensitizer for HCT 116 cells prior to treatment with **2a** are presented in Table 3. The evaluation of the 3,5-bis(benzylidene)-4-piperidones **2a** and **7a–g** against HCT 116 and CRL 1790 cells is displayed in Table 4. Several compounds in series **1** were evaluated for their effects on a number of leukemia and lymphoma cell lines, and these data are presented in Table 5. The hydrazide **1l** was examined for its ability to alter the mitochondrial membrane potential (MMP) in Ramos cells, and this result is presented in Figure 3. In addition, the effects of **1m**, **7a**, **e**, and **g** on the MMP and generation of reactive oxygen species (ROS) in HCT 116 cells are displayed in Figure 4 and Figure 5, respectively.

## 4. Discussion

The first question to be addressed is whether the compounds in series **1** and **6** possess cytotoxic properties towards various neoplasms. The three neoplastic cell lines initially chosen are colon and breast cancers, due to the high prevalence of these tumors in society. Two types of breast cancers were used—namely, MCF-7 cells, which express estrogen and progesterone receptors, as well as MDA-MB-231 cells, which have no estrogen or progesterone receptors. A greater cytotoxic potency displayed towards the MCF-7 cells than the MDA-MB-231 cells may indicate that the compounds cause toxicity, at least in part, by interacting at the estrogen and/or progesterone receptors. The hydrazides **1a**–**m** and **6a**–**c** were initially evaluated at two concentrations: 10 µM, and 100 µM. The higher concentration of 100 µM was used to find out whether the compounds had any cytotoxic properties at all, while the lower concentration of 10 µM was employed to find those compounds which inhibited the growth of the neoplasms significantly. These data are presented in Table 1.

At a concentration of 100 µM, in general, the compounds in series **1** significantly inhibit the growth of HCT 116, MCF-7, and MDA-MB-231 cells. When the concentration was lowered to 10 µM, the percentage inhibition of the growth of the tumor cell lines was reduced considerably. A number of the compounds demonstrated hormesis, i.e., a stimulating effect was observed at low concentrations followed by an inhibitory response as the concentration was raised. The compounds displaying the highest potency when 10 µM of compound was used were **1m** against HCT 116 cells, **1j**–**l** against MCF-7 neoplasms, and the only compound to inhibit the growth of MDA-MB-231 cells was **1f**. In summary, a number of these hydrazides in series **1** have antineoplastic properties, which enables them to be evaluated as potential chemosensitizers.

A comparison was made between the cytotoxic potencies of **1a** and **6a**–**c**. Both **6a** and **6b** have lower growth-inhibitory properties than **1a** towards HCT 116 and MCF-7 cells, which may be due, at least in part, to the absence of the α,β-unsaturated carbonyl group in **1a**. Somewhat surprisingly, **6c** (which is devoid of an olefinic group) has significant growth-inhibiting properties at 100 µM, and some effect at 10 µM, against HCT 116 and MCF-7 cells; in fact, it has greater potency than **1a** towards HCT 116 and MCF-7 cells. It is possible that the additional benzoylhydrazino group (NHNHCOC_6_H_5_) in **6c** compared to **6a** and **6b** causes additional interactions with cellular constituents.

The cytotoxic potencies of **1a**–**m** were compared to two reference drugs, namely, 5-fluouracil (**5-FU**) and melphalan. The antimetabolite **5-FU** was chosen because it is used extensively in treating both colon and breast cancers. Melphalan is an alkylating agent, and the α,β-unsaturated keto group present in the molecules in series **1** is capable of alkylating thiols and other cellular constituents. Comparisons of potencies were made considering the data generated using 10 µM of the compounds. The hydrazide **1m** is the most potent analog in series **1** towards HCT 116 cells, and has half of the potency of **5-FU**, but demonstrates a slightly greater potency than melphalan. In the case of MCF-7 cells, the most potent compounds are **1j**–**l**, possessing approximately half of the potency of **5-FU** and being less potent than melphalan. The most active compound towards MDA-MB-231 cells is **1f**, which has the same potency as **5-FU**, and about half the potency of melphalan.

The next step of the investigation was to identify lead compounds in terms of potency and selective toxicity for neoplasms, as well as comparing these properties with two reference drugs. These data are presented in Table 2. The evaluations utilizing HCT 116 cells will be considered first. The unsubstituted compound **1a** has an IC_50_ value of 36.9 µM, which is similar to the figure obtained for melphalan, while **1a** is less potent than **5-FU**. The IC_50_ value of 1m reveals it to be approximately twice as potent as **1a**. The absence of significant cytotoxic potencies of **6a** was confirmed. These compounds were also evaluated against human non-malignant CRL 1790 colon cells in order to generate selectivity index (SI) figures, which are the quotients of the IC_50_ value of the compound towards CRL 1790 cells divided by the IC_50_ figure against a neoplastic cell line. As indicated in Table 2, 1m has an SI value in excess of 6.54, which compares favorably with an SI figure of 2.92 for **5-FU**.

The next phase of the investigation was to determine whether a representative compound in series 1 chemosensitizes HCT 116 cells to a 3,5-bis(benzylidene)-4-piperidone **2a**. The acryloylhydrazide **1m** was chosen as it is the most potent compound in series **1**, and **2a** has been shown previously to be a potent cytotoxin [2]. The colon cancer cells were incubated for 24 h with **1m**, and then **2a** was added and the incubation was continued for a further 24 h. Different concentrations of both **1m** and **2a** were employed, and the results are presented as entries 1–12 in Table 3. In addition, the effects of each of **1m**, **2a**, and **5-FU** on HCT 116 cells (which are necessary for the evaluation of whether chemosensitivity occurs) were recorded, and are presented as entries 13–20 in Table 3.

The method of calculating whether chemosensitivity was demonstrated is as follows, using entry 4 as an example:

The concentration of **1m** in combination 4 is 5 µM, which after 48 h causes a percentage reduction of live cells to 86% (DMSO figure after 48 h, line 20) minus 75% (48 h, line 13), or 11%.

The concentration of **2a** in combination 4 is 0.2 µM. This combination leads to a reduction of the percentage of live cells to 91% (DMSO figure after 24 h, line 20) minus 69% (line 17), or 22%.

The percentage reduction in live cells caused by a combination of **1m** (5 µM) and **2a** (0.2 µM), in the absence of chemosensitization, is 11% plus 22%, or 33%.

However, the percentage reduction in cells as indicated on line 4 is 86% (DMSO figure after 48 h) minus 12% (line 4, 48 h figure), or 74%.

Thus, in this combination, chemosensitivity was demonstrated.

The conclusion drawn from the data in Table 3 is that **1m** sensitizes HCT 116 cells to **2a** when certain concentrations of both compounds are used. These data serve as proof of principle, and are a prelude to further studies to address a host of other issues, such as whether 24 h is an optimal time for chemosensitization, or whether **1m** will sensitize other cell lines to other cytotoxic agents.

A further aspect of the present study was to investigate whether combining some of the structural features of the chemosensitizer **1m** and cytotoxin **2a** would create potent antineoplastic agents with good selectivity indices. To this end, the compounds in series **7** were prepared. Reaction of **2a** with **1a** and **1m** gave rise to **7a** and **7b**, respectively, as indicated in Scheme 3. Various substituents were placed in the arylidene rings of **7b**, with a view to developing structure–activity relationships in these compounds.

The IC_50_ values of **1a** and **1m** towards HCT 116 cells are 36.9 µM and 15.3 µM, respectively, (Table 2). Hence, the analogs **7a** and **7b** are 26.7 and 4.86 times more potent than **1a** and **1m**, respectively. All of the compounds in series **7** are potent cytotoxins. In particular, **7e** is 14.7 times more potent than **5-FU**, and has 119 times the antineoplastic activity of melphalan. Furthermore **7e** has an SI value of 16.3.

An attempt was made to determine whether there were correlations between one or more of the physicochemical properties of the groups in the arylidene aryl rings of **7b**–**g** and their cytotoxic potencies. The magnitudes of the Hammett σ, Hansch π, and molar refractivity (MR) constants of these groups reflect the electronic, hydrophobic, and steric properties, respectively, of these substituents. Linear and semi-logarithmic plots were made between the IC_50_ values of **7b**–**g** in the HCT 116 and CRL 1790 screens and the σ, π, and MR values. A trend towards a negative correlation (*p* < 0.1) was noted in the semi-logarithmic plots between the IC_50_ values towards HCT 116 and the CRL1790 cells and the σ values. No other correlations (*p* > 0.1) were noted; this result suggests that potency increases as the electron-withdrawing properties of the aryl groups rise, which is somewhat ambivalent as far as drug design, since strongly attracting σ values increase potency towards both neoplasms and non-malignant cells. Hence, the next step was to find out whether there were correlations between the SI values and the σ, π, and MR constants. Both the linear and semi-logarithmic plots reveal positive correlations (*p* < 0.05) between the SI values and the σ constants. Thus, SI values were predicted to rise as electron-withdrawing groups are placed in the arylidene aryl rings of series **7**. No other correlations (*p* > 0.1) were noted. Another query to resolve is whether the IC_50_ values correlate with the SI figures, i.e., do potency increases affect SI values? Linear, semi-logarithmic, and logarithmic plots between the IC_50_ values towards HCT 116 and the CRL 1790 cells reveal negative correlations (*p* < 0.01). Thus, potency increases are accompanied by a rise in the SI values.

In summary, the cytotoxic potency of the compounds towards HCT 116 and CRL 1790 cells is likely to be influenced by electronic factors, rather than hydrophobic or steric factors. In developing these compounds further, strongly electron-withdrawing groups, such as 3-trifluoromethyl-4-nitro (∑σ = 1.21) and 3-chloro-4-nitro (∑σ = 1.15) groups, should be placed in the aryl rings [12]. 

The studies conducted so far have revealed that a number of the compounds are toxic to human tumor (HCT 116 cells) and breast cancer (MCF-7) cell lines. The decision was made to evaluate whether representative compounds are also active towards human leukemia and lymphoma cells, due to the clinical significance of these groups of cancers. The cell lines chosen were lymphoblastic leukemia (Ramos, NALM-60, Jurkat), leukemia (CEM, HL-60), and lymphoma-like (Raji) neoplasms. Four compounds were chosen for bioevaluation, namely: **1a** and **1h**, which display cytotoxicity towards both HCT 116 and MCF-7 cells; **1l**, which demonstrates good potency against MCF-7 cells; and, as a null hypothesis, **1d**, which was virtually inactive against HCT 116 and MCF-7 cells. In addition, with a view to finding compounds that display greater toxicity to neoplasms than to non-malignant cells, **1a**, **d**, **h**, and **l** were evaluated against non-malignant human Hs27 foreskin and human MCF-10A breast cells. The data generated are presented in Table 5.

The data in Table 5 reveal that **1a**, **d**, **h**, and **l** are potent cytotoxins towards the six tumor cell lines. No less than 88% of the CC_50_ values are below 5 µM, and 25% are submicromolar. In particular, Ramos cells are very sensitive to these compounds, with an average CC_50_ value of **1a**, **d**, **h**, and **l** to this neoplasm of 0.21 µM. The compounds demonstrated a greater toxicity to the neoplastic cell lines than to Hs27 and MCF-10A cells. The average SI value for **1a**, **d**, **h**, and **l** against six neoplastic cell lines is 16.0, which is noteworthy. In particular, in the case of the four compounds evaluated against Ramos cells, the average SI value rises to 66.8.

Finally an investigation was conducted in order to find out whether the antineoplastic properties and selective toxicity of the representative compounds was due, at least in part, to one or more effects on mitochondrial function. This possibility was based on the observation that differences exist between the mitochondria in certain tumors and those in some non-cancerous cells. Thus, while the mean value of the MMP in non-malignant cells has been reported to be –139 mV [14], the MMP in tumor mitochondria can be double this value [15]. In addition, the rise in the MMP is associated with an increase in the concentration of ROS [16].

In order to explore the possibility that representative compounds interfere with mitochondrial function, the following experiments were conducted: A lead compound was **1l**, which has a CC_50_ value of 0.19 µM towards Ramos cells and a huge selectivity index of 94.2 (Table 5). The MMP of Ramos cells was determined using the JC-1 dye [3,8]. In this assay, JC-1 enters the energized mitochondria in healthy cells and forms aggregates to produce red fluorescence at 590 nm. However, cells undergoing apoptotic cell death have a low mitochondrial membrane potential. For this reason, JC-1 does not form aggregates, and emits a green fluorescence that can be measured at 530 nm. The result is portrayed in Figure 3, which reveals that using the CC_50_ concentration of **1l**, the MMP is significantly depolarized compared to the control values. When the concentration of **1l** was doubled, there was an approximately twofold increase in the mitochondrial depolarization in the Ramos cells.

After this encouraging start, the decision was made to examine four of the novel compounds identified in this study for their effects on the MMP and production of ROS. The chemosensitizer **1m** was chosen along with the parent compound in series **7**, **7a**. In addition, the most potent compound with the highest SI value was **7e**, which has an electron-withdrawing group in the arylidene aryl rings. Hence, **7g** was also chosen since it has an electron-donating group in the arylidene aryl rings.

The decision was made to experiment with another method for determining the effect of compounds on the MMP. In this assay, the dye used was tetramethylrhodamine ethyl ester (TMRE), which can be found in the mitochondria of healthy cells. However, in apoptotic cells, the dye is released into the cytosol, leading to a decrease in the fluorescence signal. Using concentrations of the IC_50_ values of **1m**, **7a**, **e**, and **g** towards HCT 116 cells, the results presented in Figure 4 reveal that only **7e** reduces the electrochemical potential of the mitochondria.

The evaluation of **1m**, **7g**, **e**, and **g** for their capacity to increase the concentration of ROS in HCT 116 cells used 2′,7′-dichlorofluorescein diacetate. This dye is deacetylated in cells, and is converted into a non-fluorescent compound that reacts with ROS to form 2′,7′-dichlorofluorescein, which is fluorescent. Using the IC_50_ concentrations of **1m**, **7a**, **e**, and **g** towards HCT 116 cells, the data presented in Figure 5 reveal that all four compounds increase the fluorescence intensity in HCT 116 cells and, hence, the ROS levels in these cells. The order of potency is **7e** > **7a** > **1m** > **7g**, which suggests that, in the future, groups with electron-withdrawing properties should be placed in the arylidene aryl rings.

## 5. Conclusions

A number of *N*^2^-acryloylhydrazides display toxicity to a variety of neoplasms. The representative compound **1m** sensitizes HCT 116 cells to the potent cytotoxin **2a,** and also to the established anticancer drug **5-FU**. This finding serves as a stimulus to further studies to discover the extent to which 1m can sensitize other neoplastic cells. Furthermore, the timing of the addition of the sensitizer and cytotoxin needs to be investigated. The syntheses and bioevaluations of series **7** are promising, and analog development should be undertaken to find compounds with increased potency and drug likeness. The greater toxicity for neoplasms may be due, at least in part, to a larger inhibitory effect on the mitochondria in tumors than in non-malignant cells.

## Figures and Tables

**Table 1:** The percentage inhibition of HCT 116, MCF-7, and MDA-MB-231 cells by **1a–m** and **6a–c.**

Compound	HCT 116		MCF-7		MDA-MB-231	
	10 µM	100 µM	10 µM	100 µM	10 µM	100 µM
**1a**	10.6 ± 1.70	94.1 ± 1.44	5.47 ± 3.52	51.1 ± 2.87	−12.2 ± 1.50	52.1 ± 2.04
**1b**	−2.35 ± 2.24	60.7 ± 3.22	−40.1 ± 3.34	33.2 ± 3.13	−7.37 ± 1.52	39.9 ± 2.51
**1c**	−3.28 ± 1.50	87.9 ± 0.91	−4.68 ± 3.17	46.6 ± 3.35	−16.5 ± 1.45	88.7 ± 1.26
**1d**	−5.05 ± 2.36	82.3 ± 0.80	3.59 ± 2.08	55.9 ± 2.82	−12.3 ± 4.05	49.1 ± 2.04
**1e**	4.30 ± 1.38	75.7 ± 1.65	8.66 ± 1.81	75.8 ± 2.76	−7.15 ± 2.29	91.9 ± 4.53
**1f**	13.5 ± 1.79	95.7 ± 1.71	−15.5 ± 3.38	85.9 ± 2.70	15.6 ± 3.14	98.7 ± 0.92
**1g**	6.72 ± 1.97	>100	−50.5 ± 3.27	97.4 ± 2.60	−19.4 ± 3.01	139.8 ± 1.03
**1h**	7.12 ± 1.51	87.7 ± 1.62	14.7 ± 3.68	54.2 ± 3.42	−5.95 ± 4.21	122.3 ± 2.90
**1i**	15.43 ± 2.13	93.9 ± 3.35	−82.8 ± 3.48	44.2 ± 2.96	−5.91 ± 3.86	53.3 ± 2.89
**1j**	10.4 ± 1.61	62.1 ± 2.48	53.3 ± 3.07	>100	−2.64 ± 2.37	46.8 ± 3.63
**1k**	11.5 ± 2.99	>100	55.3 ± 3.05	>100	−8.78 ± 1.52	74.7 ± 2.96
**1l**	8.62 ± 2.79	85.1 ± 3.28	60.6 ± 3.29	99.4 ± 2.34	−4.95 ± 3.12	47.7 ± 3.19
**1m**	46.9 ± 2.55	97.9 ± 1.92	−16.1 ± 3.51	>100	−15.89 ± 1.96	50.5 ± 1.42
**6a**	−10.5 ± 2.93	9.35 ± 2.53	−19.6 ± 3.66	−5.69 ± 2.54	−25.3 ± 1.82	−11.6 ± 2.52
**6b**	−4.89 ± 2.52	13.6 ± 3.03	−93.8 ± 4.15	−58.7 ± 3.24	−22.6 ± 2.65	−15.3 ± 3.46
**6c**	18.6 ± 3.98	96.9 ± 1.86	16.1 ± 3.55	97.9 ± 2.68	−16.0 ± 2.91	95.6 ± 4.22
**5-FU**	84.7 ± 2.53	94.1 ± 0.96	91.2 ± 3.03	99.3 ± 2.87	17.4 ± 2.97	>100
Melphalan	33.9 ± 1.81	98.7 ± 1.12	>100	>100	28.6 ± 2.48	>100

**Table 2:** Quantitative evaluation of the cytotoxicity of selected compounds.

Compound	Neoplastic Cells		Non-Malignant CRL 1790 Cells	SI ^a^
	Cell Line	IC_50_ (µM)	IC_50_ (µM)	
**1a**	HCT 116	36.9 ± 2.84	>100	>2.70
**1m**	HCT 116	15.3 ± 0.42	>100	>6.54
**6a**	HCT 116	>100	>100	~1.00
**5-FU**	HCT 116	3.77 ± 0.48	10.9 ± 1.43	2.92
Melphalan	HCT 116	30.7 ± 2.73	>100	>3.25
**1j**	MCF-7	9.14 ± 1.31	>100	>10.9
**1k**	MCF-7	5.54 ± 0.51	>100	>18.1
**1l**	MCF-7	3.18 ± 0.53	>100	>31.5
**5-FU**	MCF-7	2.00 ± 0.25	10.8 ± 1.64	5.42
Melphalan	MCF-7	12.0 ± 1.43	>100	>8.32
**5-FU**	MDA-MB-231	15.9 ± 1.65	10.8 ± 1.64	0.68
Melphalan	MDA-MB-231	52.9 ± 2.32	>100	>1.90

^a^ The letters SI refer to the selectivity index figures, i.e., the IC_50_ values of the compounds for CRL 1790 cells divided by the IC_50_ figures for neoplastic cells.

**Table 3:** Evaluation of combinations of **1m** and **2a** for chemosensitization of the growth of HCT 116 cells.

Entry	Compounds	Live Cells %		Reduction in Live Cells %		Cal ^a^	Found ^b^	*p* Value
		24 h	48 h	1m (48 h)	2a (24 h)			
1	**1m** 5 µM + **2a** 0.1 µM	70 ± 2.13	63 ± 0.35	11	8	19	23	>0.05
2	**1m** 10 µM + **2a** 0.1 µM	68 ± 1.82	59 ± 1.99	29	8	37	27	>0.05
3	**1m** 15 µM + **2a** 0.1 µM	55 ± 0.71	45 ± 3.32	54	8	62	41	<0.05
4	**1m** 5 µM + **2a** 0.2 µM	39 ± 1.19	12 ± 0.18	11	22	33	74	<0.05
5	**1m** 10 µM + **2a** 0.2 µM	27 ± 0.21	10 ± 0.26	29	22	51	76	<0.05
6	**1m** 15 µM + **2a** 0.2 µM	17 ± 1.48	0	54	22	76	100	<0.05
7	**1m** 5 µM + **2a** 0.4 µM	19 ± 1.25	0	11	43	54	100	<0.05
8	**1m** 10 µM + **2a** 0.4 µM	11 ± 1.40	0	29	43	72	100	<0.05
9	**1m** 15 µM + **2a** 0.4 µM	5 ± 0.29	0	54	43	97	100	>0.05
10	**1m** 5 µM + **5-FU** 3 µM	53 ± 2.39	37 ± 1.73	11	13	24	49	<0.05
11	**1m** 10 µM + **5-FU** 3 µM	50 ± 1.21	32 ± 0.43	29	13	42	54	>0.05
12	**1m** 15 µM + **5-FU** 3 µM	32 ± 1.48	20 ± 0.55	54	13	67	66	>0.05
13	**1m** 5 µM	88 ± 2.82	75 ± 4.89	--	--	--	--	--
14	**1m** 10 µM	67 ± 1.67	57 ± 1.76	--	--	--	--	--
15	**1m** 15 µM	61 ± 3.49	32 ± 0.94	--	--	--	--	--
16	**2a** 0.1 µM	83 ± 2.28	78 ± 2.12	--	--	--	--	--
17	**2a** 0.2 µM	69 ± 1.38	52 ± 1.15	--	--	--	--	--
18	**2a** 0.4 µM	48 ± 1.20	32 ± 1.50	--	--	--	--	--
19	**5-FU** 3 µM	78 ± 2.44	55 ± 1.37	--	--	--	--	--
20	DMSO	91 ± 1.30	86 ± 0.65	--	--	--	--	--

^a^ The calculated values are the sum of the percentage reduction of live cells caused by **1m** after 48 h and either **2a** or **5-FU** after 24 h. ^b^ The found values are the percentage reduction of live cells after 48 h of the combinations of **1m** and either **2a** or **5-FU**. Three different independent experiments were conducted with three replicates in each experiment.

**Table 4:** Evaluation of **2a** and **7a–g** against HCT 116 and CRL 1790 cells.

Compound	Aryl Substituent			IC_50_ (µM)		SI ^a^
	R^1^	R^2^	R^3^	HCT 116	CRL 1790	
**7a**	H	H	H	1.38 ± 0.25	12.3 ± 1.09	3.87
**7b**	H	3-OCH_3_	4-OCH_3_	3.15 ± 0.33	9.63 ± 1.62	3.06
**7c**	4-Cl	3-OCH_3_	4-OCH_3_	3.40 ± 0.01	12.2 ± 1.22	3.60
**7d**	4-F	3-OCH_3_	4-OCH_3_	3.82 ± 0.11	11.4 ± 2.22	2.99
**7e**	4-NO_2_	3-OCH_3_	4-OCH_3_	0.28 ± 0.01	4.57 ± 0.53	16.3
**7f**	4-OCH_3_	3-OCH_3_	4-OCH_3_	2.87 ± 0.23	11.1 ± 0.52	3.86
**7g**	4-CH_3_	3-OCH_3_	4-OCH_3_	1.94 ± 0.18	9.24 ± 0.52	4.76
**2a**	--	--	--	0.37 ± 0.06	13.5 ± 1.99	36.4
**5-FU**	--	--	--	4.11 ± 0.22	10.8 ± 1.64	2.64
Melphalan	--	--	--	33.2 ± 1.70	>100	>3.02

^a^ The letters SI refer to the selectivity index, i.e., the quotients of the IC_50_ values of the compound for CRL 1790 cells divided by the IC_50_ figures of the compound for HCT 116 cells.

**Table 5:** Evaluation of **1a**, **1d**, **1h,** and **1l** against a number of neoplastic and non-malignant cell lines.

Compound	Neoplastic Cells, CC_50_ (µM) and SI ^a^							Non-Malignant Cells, CC_50_ (µM)		
	Ramos	NALM-60	CEM	HL-60	JURKAT	RAJI	Avg	Hs27	MCF10A	Avg
**1a**	0.19 ± 0.01 (67.9)	2.74 ± 0.59 (4.71)	0.91 ± 0.48 (14.2)	3.32 ± 0.26 (3.89)	3.52 ± 0.36 (3.65)	5.18 ± 0.35 (2.49)	2.64 (16.1)	12.1 ± 0.91	13.7 ± 1.10	12.9
**1h**	0.29 ± 0.05 (47.2)	3.34 ± 0.43 (4.10)	3.37 ± 0.18 (4.07)	5.62 ± 0.29 (2.44)	2.50 ± 0.24 (5.48)	5.50 ± 1.10 (2.49)	3.44 (11.0)	14.4 ± 0.24	12.9 ± 0.95	13.7
**1d**	0.15 ± 0.02 (57.9)	2.86 ± 0.55 (3.04)	0.86 ± 0.07 (10.1)	2.90 ± 0.76 (3.00)	1.07 ± 0.09 (8.12)	2.71 ± 0.09 (3.24)	1.76 (14.2)	10.5 ± 0.05	6.87 ± 0.48	8.69
**1l**	0.19 ± 0.01 (94.2)	3.25 ± 0.99 (5.51)	1.42 ± 0.25 (12.6)	1.86 ± 0.66 (9.62)	2.17 ± 0.45 (8.25)	2.93 ± 0.43 (6.11)	1.97 (22.7)	17.7 ± 0.62	18.1 ± 0.84	17.9
Avg CC_50_ (µM)	0.21	3.05	1.64	3.43	2.32	4.08	2.45	13.7	12.9	13.3

^a^ The letters SI refer to the selectivity index, which is obtained by dividing the average CC_50_ value of non-malignant cells by the CC_50_ figure of each compound against a specific cell line. This results are in parentheses beneath the CC_50_ values.

## Data Availability

Not applicable.

## Supplementary Materials

The following are available online at https://www.mdpi.com/article/10.3390/medicines8060027/s1, S1: Synthesis of the Esters **4a-m**, S2: Synthesis of the Hydrazides **5a-m**, S3: Some Physical Data of **2a-f**, S4: Structures of Melphalan and 5-flurouracil.
