# Supplementary material for: Design, Syntheses, and Bioevaluations of Some Novel N2-Acryloylbenzohydrazides as Chemostimulants and Cytotoxic Agents
Source: Medicines (Basel). 2021 Jun 3;8(6):27. doi: 10.3390/medicines8060027 (PMC8226474; doi:10.3390/medicines8060027)
Supplement: Supplementary file 1 [file medicines-08-00027-s001.zip › medicines-1240118 Supp.M2.pdf]

Supplementary Materials: Design, Syntheses and Bioevaluations of Some Novel *N*²-Acryloylbenzohydrazides as Chemostimulants and Cytotoxic Agents

Kinjal Lakhani, Edgar A. Borrego, Karla G. Cano, Jonathan R. Dimmock, Renato J. Aguilera, Swagatika Das, Praveen K. Roayapalley, Rajendra K. Sharma and Umashankar Das

S1 Synthesis of the Esters 4a-m

A mixture of a substituted or unsubstituted benzoic acids 3a-m (0.0082 mol), ethanol (0.085 mol, 5 mL) and sulfuric acid (0.0094 mol, 0.5 mL) was heated under reflux for 6–8 h [1].The reaction was monitored by TLC using a solvent system of hexane and ethyl acetate (1:1). In case of 4g and 4m, the esters precipitated from the reaction mixture. In other cases, the solvent was removed *in vacuo* to give the crude esters. All of the compounds were dissolved in ethyl acetate (20 mL) and washed twice with saturated sodium bicarbonate solution (20 mL). The organic phase was separated, washed with brine (20 mL) and dried over sodium sulfate. Removal of ethyl acetate yielded the desired esters. All the compounds in series 4 were identified by ^{1}H NMR and the data for three representative compounds are given below. Table S1 indicates some physical data of these esters.

Ethyl benzoate (4a): Colorless liquid; Yield = 83%; ^{1}H NMR ($CDCl_3$): δ 8.08 (dd, J = 8.32 Hz, 1.31 Hz, 2H, Ar-H), 7.54 (m, 1H, Ar-H), 7.45 (m, 2H, Ar-H), 4.40 (q, J = 14.25 Hz, 7.12 Hz, 2H, CH_2), 1.39 (t, J = 7.12 Hz, J = 14.23 Hz, 3H, CH_3).

Ethyl 3,4-dichlorobenzoate (4g): Colorless crystals; Yield = 76%; m.p. 37 °C (lit. m.p. 35–37 °C) [2]; ^{1}H NMR ($CDCl_3$): δ 8.12 (d, J = 1.92 Hz, 1H, Ar-H), 7.87 (dd, J = 8.36 Hz, 1.95 Hz, 1H, Ar-H), 7.52 (d, J = 8.38 Hz, 1H, Ar-H), 4.40 (q, J = 14.25 Hz, 7.16 Hz, 2H, CH_2), 1.40 (t, J = 7.17 Hz, J = 14.33 Hz, 3H, CH_3).

Ethyl 3,4-dimethoxybenzoate (4m): Colorless crystals; Yield = 71%; m.p. 42–44 °C (lit. m.p. 43–44 °C) [3]; ^{1}H NMR ($CDCl_3$): δ 7.69 (dd, J = 8.42 Hz, 1.75 Hz, 1H, Ar-H), 7.55 (d, J = 1.63 Hz, 1H, Ar-H), 6.88 (d, J = 8.46 Hz, 1H, Ar-H),4.36 (q, J = 14.28 Hz, 7.19 Hz, 2H, $CH_{2,}$), 3.94 (s, 6H, 2x$OCH_{3,}$), 1.39 (t, J = 7.13 Hz, J = 14.27 Hz, 3H, CH_3).

Table S1. Some physical data of 4a-m.

Compounds	Substituents	Appearance	m.p. (°C)	Lit. m.p (°C)	Yield (%)
4a	H	Colorless liquid	--	--	83
4b	4-F	Slightly yellow liquid	--	--	76
4c	4-Br	Slightly yellow liquid	--	--	52
4d	4-Cl	Colorless liquid	--	--	78
4e	2-Cl	Slightly yellow liquid	--	--	78

4f	3-Cl	Slightly yellow liquid	--	--	85
4g	3,4-Cl_2	Colorless solid crystals	37–39	35–37 [2]	76
4h	2,5-Cl_2	Slightly red brown liquid	--	--	75
4i	3-CH_3	Yellow liquid	--	--	73
4j	2-CH_3	Yellow liquid	--	--	81
4k	4-CH_3	Colorless liquid	--	--	89
4l	4-OCH_3	Slightly yellow liquid	--	--	86
4m	3,4-$(OCH_3)_2$	Colorless solid crystals	42–44	43–44[3]	71

S2 Synthesis of the Hydrazides 5a-m

A solution of hydrazine hydrate in water (50–60%, 1.67 mL, 33.3 mmol) and a solution of the ethyl benzoate (1 g, 66.6 mmol) were mixed and then heated under reflux for 8–12 h [4].The reaction was monitored by TLC using a solvent system of chloroform and methanol (9.5:0.5). After the reaction was completed, the solvents were removed *in vacuo* and the product recrystallized from ethanol (95%). These compounds have ^{1}H NMR spectra consistent with the structures proposed and the data for three representative compounds are given below. Some physical data for the compounds in series 5 are presented in Table S2.

Benzohydrazide (5a): Colourless crystals; Yield = 75%; m.p.110°C (lit. m.p. 112 °C) [1];^{1}H NMR (500 MHz, DMSO-d_6) δ ppm 9.76 (s, 1H, CONH), 7.81 (d, J = 7.87 Hz, 2H, Ar-H), 7.51 (t, J = 6.9 Hz, J = 13.78 Hz, 1H, Ar-H), 7.44 (t, J = 7.6 Hz, J = 15.17 Hz, 2H, Ar-H), 4.48 (s, 2H, NH_2).

3,4-Dicholorbenzohydrazide (5g): Colourless crystals; Yield = 93%; m.p. 167–170 °C (lit. m.p. 154–166 °C) [2];^{1}H NMR (500 MHz, DMSO-d_6) δ ppm 9.96 (s, 1H, CONH), 8.04 (d, J = 1.94, 1H, Ar-H), 7.80 (dd, J = 8.37 Hz, 1.98 Hz, 1H), 7.74 (d, J = 8.36 Hz, 1H, Ar-H), 4.55 (s, 2H, Ar-H).

3,4-Dimethoxybenzohydrazide (5m): Colourless crystals; Yield = 91%; m.p. 148–150 °C (lit. m.p. 151-152 °C) [5];^{1}H NMR (500 MHz, DMSO-d_6) δ ppm 9.63 (s, 1H, CONH), 7.43 (td, 2H, Ar-H), 7.00 (d, J = 8.38 Hz, 1H, Ar-H), 4.47 (s, 2H, Ar-H), 3.79 (d, J = 1.85 Hz, 6H, 2xOCH_3).

Table S2. Physical data of 5a-m.

Compounds	Substituents	Appearance	m.p. (°C)	Lit. m.p (°C)	Yield (%)
5a	H	Colorless crystals	110–112	112 [1]	95
5b	4-F	Slightly yellow crystals	160–162	162 [1]	69
5c	4-Br	Colorless crystals	166–168	164 [1]	42
5d	4-Cl	Colorless crystals	159–162	163 [1]	66
5e	2-Cl	Colorless crystals	118–121	118–120 [6]	74
5f	3-Cl	Colorless crystals	154–157	158 [1]	93
5g	3,4-Cl_2	Colorless crystals	167–170	154–166 [2]	73
5h	2,5-Cl_2	Colorless crystals	167–172	168–170 [2]	35

5i	3-CH_3	Colorless crystals	99–102	97 [1]	88
5j	2-CH_3	Colorless crystals	118–120	124 [1]	83
5k	4-CH_3	Yellow crystals	115–118	117 [1]	90
5l	4-OCH_3	Slightly yellow crystals	134–136	136–140 [6]	88
5m	3,4-$(OCH_3)_2$	Colorless crystals	148–150	151–152 [5]	91

S3 Some Physical Data of 2a-f

3,5-Bis(benzylidene)-4-piperidone 2a: Yield = 86%; m.p. 175–177 °C (lit. m.p.177–178 °C) [7]; ^{1}H NMR (500 MHz, DMSO-d_6) δ ppm 7.82 (brs, 2H, =CH), 7.40 (m, 10H, Ar-H), 4.16 (d, J= 1.73 Hz, 4H, piperidyl H). The NH peak was not observed.

3,5-Bis(4-Chlorobenzylidene)-4-piperidone 2b: Crude yield = 64%; ^{1}H NMR (500 MHz, DMSO-d_6) δ ppm 7.55 (brs, 2H, =CH), 7.52 (s, 8H, Ar-H), 3.96 (d, J = 1.33, 4H, piperidyl H). The NH peak was not observed.

3,5-Bis(4-Fluorobenzylidene)-4-piperidone 2c: Crude yield = 40%; ^{1}H NMR (500 MHz, DMSO-d_6) δ ppm 7.56 {d, J = 8.94 Hz, 6H (4H, Ar-H; 2H, =CH)}, 7.30 (m, 4H, Ar-H), 3.96 (s, 4H, piperidyl H). The NH peak was not observed.

3,5-Bis(4-Nitrobenzylidene)-4-piperidone 2d: Crude yield = 94%; ^{1}H NMR (500 MHz, DMSO-d_6) δ ppm 8.28 (d, J = 7.48 Hz, 4H, Ar-H), 7.76 (d, J = 7.57 Hz, 4H, Ar-H), 7.66 (s, 2H, =CH), 4.02 (s, 4H, piperidyl H). The NH peak was not observed.

3,5-Bis(4-Methyoxybenzylidene)-4-piperidone 2e: Crude yield = 73%; ^{1}H NMR (500 MHz, DMSO-d_6) δ ppm 7.53 (s, 2H, =CH), 7.44 (s, 4H, Ar-H), 7.02 (s, 4H, Ar-H), 3.96 (s, 4H, piperidyl H), 3.8 (s, 6H, 2xOCH_3). The NH peak was not observed.

3,5-Bis(4-Methylbenzylidene)-4-piperidone 2f: Crude yield = 72%; ^{1}H NMR (500 MHz, DMSO-d_6) δ ppm 7.66 (brs, 2H, =CH), 7.40 (d, 4H, J = 7.85 Hz, Ar-H), 7.30 (d, 4H, J = 7.77 Hz, Ar-H), 4.15 (s, 4H, piperidyl H), 2.35 (s, 3H, CH_3). The NH peak was not observed.

S4 Structures of Melphalan and 5-flurouracil

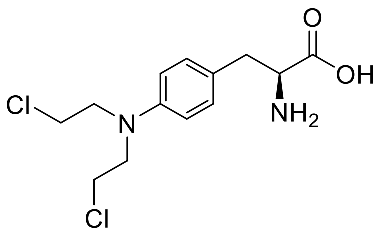

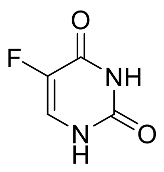

References

1. Furniss, B.S.; Hannaford, A.J.; Smith, P.W.; Tatchell, A.R. Vogel's Textbook of Practical, *Organic Chemistry*; Longman Scientific and Technical, Essex England, 1989; pp 1077–1078.
2. Blickenstaff, R.T.; Hanson, W.R.; Reddy,S.; Witt, R. Potential Radioprotective Agents-VI. Chalcones, Benzophenones, Acid Hydrazides, Nitro Amines and Chloro Compounds. Radioprotection of Murine Intestinal Stem Cells. *Bioorg. Med. Chem*. **1995**, *3*, 917–922.
3. Available at : https://www.chemicalbook.com/ProductChemicalPropertiescb3406135_EN.htm.

4. Yale, H.L.; Losee, K.; Martins, J.; Holsing, M.; Perry, F.M.; Bernstein, J. Chemotherapy of Experimental Tuberculosis. VIII. The Synthesis of Acid Hydrazides, Their Derivatives and Related Compounds. *J. Amer. Chem. Soc.* **1953**, *75*, 1933–1942.
5. Saha, A.; Kumar, R.; Kumar, R.; Devakumar, C. Development and Assessment of Green Synthesis of Hydrazides. *Ind. J. Chem*. 2010, *49*B, 526–531.
6. Kaushik, D.; Khan, S.A.; Chawla, G.; Kumar, S. N′-[(5-Chloro-3-Methyl-1-Phenyl-1H-Pyrazol-4-Yl)Methylene] 2/4-Substituted Hydrazides: Synthesis and Anticonvulsant Activity. *Eur. J. Med. Chem*. **2010**, *45*, 3943–3949.
7. Dimmock, J.R.; Padmanilayam, M.P.; Puthucode, R.N.; Nazarali, A.J.; Motaganahalli, N.L.; Zello, G.A.; Quail, J.W.; Oloo, E.O.; Kraatz, H.B.; Prisciak, J.S.; et al. A Conformational and Structure-Activity Relationship Study of Cytotoxic 3,5-Bis(Arylidene)-4-Piperidones and Related N-Acryloyl Analogues. *J.Med. Chem.* **2001**, *44*, 586–593.
